# The Value of Pressure Indexes in Predicting the Outcome of Diabetic Foot Ulcers as a Guide to Further Management

**DOI:** 10.7759/cureus.69164

**Published:** 2024-09-11

**Authors:** Rushabh A Parekh, Virendra Athavale, Saili Kelshikar

**Affiliations:** 1 Department of General Surgery, Dr. D. Y. Patil Medical College, Hospital and Research Centre, Dr. D. Y. Patil Vidyapeeth (Deemed to be University), Pune, IND

**Keywords:** abpi, diabetes foot, diabetes mellitus, leg doppler, peripheral vascular

## Abstract

Introduction: Diabetic foot ulcers (DFUs) stand out as a prevalent and debilitating condition. Furthermore, the progression of diabetes-related hyperglycemia and associated conditions such as peripheral arterial disease exacerbates the risks of DFUs, including complications like infection and gangrene. Given the significant burden of DFUs, there is a pressing need to improve preventive and therapeutic strategies. This study investigates the clinical profiles of patients with DFUs, including the importance of smoking and intermittent claudication, evaluates management strategies based on the vascular status assessed through Doppler scans, and provides insights into the importance of ankle-brachial pressure index (ABPI) and toe brachial index (TBI) values, which play a vital role in predicting and managing ulcer healing outcomes, highlighting the importance of vascular assessment in treatment planning.

Aim: The study aims to assess the clinical profile of patients with DFUs based on their vascularity status using Doppler scans, predict the outcome of the ulcer using indexes like ABPI and TBI values, and decide on further management.

Materials and methods: This prospective study was conducted over 24 months, from August 2022 to July 2024, at the Department of General Surgery, Dr. D. Y. Patil Medical College, Hospital and Research Centre, Pimpri, Pune. The study included 50 patients presenting with DFUs. Approval was obtained from the Institutional Ethics Committee, and informed consent was obtained from all participants. A detailed history was taken, including the history of smoking and intermittent claudication, and a thorough examination of the ulcer was performed. HbA1c levels were measured, Doppler scans of the lower limbs were done, and the ABPI and TBI values were calculated. Management strategies were determined based on the assessed vascular status, leading to either medical or surgical interventions.

Results: Twenty-one (42%) patients who presented with DFUs had a positive history of intermittent claudication, demonstrating a significant association between the history of intermittent claudication and the Doppler findings. Thirty-seven (74%) patients gave a positive history of smoking, which revealed a significant association between the history of smoking and Doppler findings. Thirty-eight (76%) patients had an ABPI of <0.9, out of which 33 (66%) patients showed an improved outcome of the ulcer after necessary management. There is also a statistically significant association between the ABPI and Doppler findings. Sixteen (32%) of patients had a TBI of <0.65, of which all showed an improvement. There is a highly significant association between TBI and the Doppler findings.

Conclusion: This study concludes that integrated routine assessment of ABPI and TBI into DFU management protocols is necessary to guide treatment decisions and monitor response to therapy. Treatment and prevention of diabetes-related complications affecting the lower extremities require a dedicated interdisciplinary approach.

## Introduction

Diabetes mellitus (DM) is a prevalent medical ailment with a greater incidence in developing nations. It significantly affects the quality of life because of its acute and long-term problems resulting from the involvement of micro- and microvasculatures [1].

Globally, DM and its consequences are a leading cause of morbidity and mortality. Glycemic control has a direct impact on the onset and course of chronic diabetic complications. One of the most common causes of hospitalization in a surgical ward is a diabetic foot ulcer (DFU); complications from this condition can include abscesses, cellulitis, ulcers, and gangrene. Since ulcers are the primary cause of lower limb amputation in diabetics, it is crucial to treat them. Clinicians must discover newer techniques to prevent ulcerations. The incidence and morbidity of DFUs can be significantly decreased using evidence-based preventive and treatment methods [2,3].

Up to 70% of lower limb amputations are due to diabetes. Ulcers and diabetic foot disease cause the majority of diabetic individuals to need amputations. The burden of diabetic foot illness and ulceration is increased by peripheral neuropathy and peripheral artery disease (PAD). PAD accounts for over 60% of DFUs because of inadequate blood supply. PAD is a disorder marked by occlusive alterations in the arteries supplying the lower limbs. It has been shown that compared to nondiabetic patients, peripheral ischemia event rates are greater in diabetic patients. In those with DM, it is a significant contributing factor to lower extremity amputation due to DFUs and gangrene [4].

The two main underlying causes of PAD are reported to be peripheral neuropathy and ischemia. Even mild ischemia can impede healing and result in ulcers when these conditions are met. DFUs fall into one of two categories: neuroischemic ulcers (NIUs) or neuropathic ulcers, each with a distinct prognosis and course of therapy [5].

Men and women with diabetes have greater rates of PAD. Symptoms like intermittent claudication, discomfort during exercise, pain at rest, and, in extreme situations, critical limb ischemia and gangrene are all directly related to PAD. PAD is diagnosed by intermittent claudication and the significant impairment or absence of at least one main arterial pulse. Age, length of diabetes, and males with diabetes all correlate with an increase in PAD prevalence. In the general population, smoking, excessive cholesterol, tobacco use, and hypertension all contribute to PAD. In diabetics, PAD increases the risk of lower limb ulcers, gangrene, abscesses, and amputations. Foot ulcers affect 15% of diabetic patients. Hospitalization may be necessary to manage foot ulcer complications [6,7].

Ischemia brought on by atherosclerosis may worsen due to microvascular disease. Many studies have been conducted on the connection between DFUs and PAD. In addition to impaired ulcer healing, poor arterial inflow is linked to infection, gangrene, and amputation. A rising number of DFUs in developed countries are caused by ischemia [4].

The management of infection by minor amputation and/or drainage occurs first, followed by an evaluation for limb ischemia and, finally, vascular restoration, such as a distal bypass to the lower leg arteries. The main goal of managing diabetic foot illness is to prevent amputation of the lower limbs. When DFUs are treated aggressively and promptly, it is frequently possible to avoid both an escalation of the condition and the possibility of amputation. Therefore, the goal of therapy should be early intervention to enable the lesion to heal quickly and, once healed, to avoid its recurrence. Clinicians need to be aware of these tactics to save diabetic foot patients. Since only ischemic individuals require revascularization, these categories aid in the diagnosis of both ischemia and nonischemic foot [7].

Treatment and prevention of diabetes-related complications affecting the lower extremities require a dedicated interdisciplinary approach. The current study aims to investigate the clinical profile of patients suffering from DFUs. The management strategies for these patients were determined after the vascularization status was assessed using a variety of imaging modalities, including Doppler scans. Depending on the patient's lower limb vasculopathy condition, the DFUs were managed accordingly.

## Materials and methods

Aim

The study's aim was to evaluate the clinical profile of patients with DFUs, assess the vascularity status of the DFUs using Doppler scans, and predict the outcome of the ulcers using indexes like the ankle-brachial pressure index (ABPI) and toe brachial index (TBI) values to decide on further management.

Study design

This prospective study was conducted in the Department of General Surgery at Dr. D. Y. Patil Medical College, Hospital and Research Centre, Pimpri, Pune. The duration of the study was from August 2022 to July 2024. The sampling technique used was purposive sampling, with a sample size of 50 patients with DFUs. The sample size calculation method was n = z^2^ × P (100 - P)/d^2^. Ethics approval from the Research and Recognition Committee under the Faculty of Medicine of Dr. D. Y. Patil Medical College, Hospital and Research Centre was taken before the start of the study (I.E.S.C./PGS/2022/76). Every participant gave their informed consent.

The study included all proven cases of DFUs with type 2 DM (T2DM), aged 25-75. Patients with type 1 DM, those aged less than 25 and more than 75, and those not willing to give consent were excluded from the study.

Patients who had presented with DFUs were diagnosed by checking blood sugar levels, previous history of being a known case of DM, and HbA1c levels. Their detailed history, along with the history of intermittent claudication and smoking, was taken. An examination of the ulcer was done along with inspection of the limbs to check for blackening, loss of hair, shiny skin, and lipodermatosclerosis. Palpation of the dorsalis pedis artery, anterior tibial artery, posterior tibial artery, popliteal artery, and femoral artery was done. A single radiologist did Doppler scans of the lower limbs to assess the vascular status of the patient's limbs, and ABPI and TBI values were calculated. The dimensions of the ulcer were taken at the time of admission and then taken again at the end of three months. Computed tomography (CT), along with angiography of the lower limbs, was done to assess the stenosis or occlusion of the arteries of the lower limb in a few cases. The ulcers were managed through a process involving regular dressings, debridement, and an assessment of the vascular status. This was followed by the appropriate treatment, either split-thickness skin grafting or amputation, based on the limb's vascular status.

Descriptive statistics were presented in the form of percentages. Statistical analysis was done using Epi-7 or open EPI (Centers for Disease Control and Prevention, Atlanta, GA). The statistical package used was WinPEPI (updated by J. H. Abramson). The cutoff value for significance was at p < 0.05.

## Results

Most individuals in the study fell within the age range of 41-50 years, with a mean age of 50.72 years and a standard deviation of 9.54. The age distribution ranged from 34 to 73 years, with a total sample size of 50 individuals. The study evaluated the outcomes of 50 individuals based on gender, with 23 females (46%) and 27 males (54%).

In Table 1, the history of intermittent claudication was present in 21 (42%) patients. The analysis indicates a significant association between the history of intermittent claudication and the Doppler findings at p < 0.05.

**Table 1:** Association of history of intermittent claudication with Doppler findings The chi-square statistic is 29.4675. The p value is <0.00001 The result is significant at p < 0.05

History of intermittent claudication	Monophasic, n (%)	Biphasic, n (%)	Triphasic, n (%)	Total, n (%)
Yes	14 (28)	5 (10)	2 (4)	21 (42)
No	1 (2)	4 (8)	24 (48)	29 (58)
Total	15 (30)	9 (18)	26 (52)	50 (100)

Table 2 reveals a significant association between smoking history and Doppler findings (monophasic, biphasic, and triphasic). This finding underscores the importance of smoking history and its impact.

**Table 2:** Association of history of smoking and Doppler findings The chi-square statistic is 13.6086. The p value is 0.001109 The result is significant at p < 0.05

History of smoking	Monophasic, n (%)	Biphasic, n (%)	Triphasic, n (%)	Total, n (%)
No	6 (12)	7 (14)	24 (48)	37 (74)
Yes	9 (18)	2 (4)	2 (4)	13 (26)
Total	15 (30)	9 (18)	26 (52)	50 (100)

In Table 3, the chi-square statistic of 15.812 with a p value of 0.000369 suggests a statistically significant association, at p < 0.05, between the presence of other comorbidities and the Doppler findings.

**Table 3:** Association of other comorbidities with Doppler findings

Other comorbidities	Monophasic, n (%)	Biphasic, n (%)	Triphasic, n (%)	Total, n (%)
Bronchial asthma	1 (2)	1 (2)	1 (2)	3 (6)
Hypertension	9 (18)	2 (4)	1 (2)	12 (24)
None	5 (10)	6 (12)	24 (48)	35 (70)
Total	15 (30)	9 (18)	26 (52)	50 (100)

In Table 4, the study investigated the relationship between ABPI and ulcer outcomes among 50 individuals. The mean ABPI was 0.86, with a standard deviation of 0.21, ranging from 0.4 to 1.4. The ABPI values were categorized as <0.9 and >0.9. Among the individuals, 38 (76%) had ABPI values of <0.9, with 33 (66%) showing improved outcomes and five (10%) remaining unchanged after necessary management. A total of 12 individuals (24%) had ABPI values of >0.9, with seven (14%) showing improved outcomes and five (10%) remaining unchanged. The chi-square statistic is 9.3771, and the p value is 0.002197, indicating that the results are statistically significant at p < 0.05.

**Table 4:** ABPI with outcome of ulcer after three months ABPI: ankle-brachial pressure index

ABPI	Improved outcome, n (%)	Unchanged outcome, n (%)	Total, n (%)
<0.9	33 (66)	5 (10)	38 (76)
>0.9	7 (14)	5 (10)	12 (24)
Total	40 (80)	10 (20)	50 (100)
Mean	0.86		
Standard deviation	0.21		
Maximum	1.4		
Minimum	0.4		

In Table 5, the chi-square statistic of 7.5448 with a p value of 0.022997 indicates a statistically significant association between the ABPI and Doppler findings since the p value is less than the significance level of 0.05.

**Table 5:** Association of ABPI with Doppler findings ABPI: ankle-brachial pressure index

ABPI	Monophasic, n (%)	Biphasic, n (%)	Triphasic, n (%)	Total, n (%)
<0.9	15 (30)	8 (16)	15 (30)	38 (76)
>0.9	0 (0)	1 (2)	11 (22)	12 (24)
Total	15 (30)	9 (18)	26 (32)	50 (100)

The study analyzed the impact of TBI on ulcer outcomes among 50 individuals. Table 6 categorized the TBI values as ≥0.65 and <0.65. Out of 30 individuals (60%) with normal TBI (≥0.65), 20 showed improved outcomes, and 10 remained unchanged. Among 17 individuals (34%) with low TBI (<0.65), all showed improved outcomes. Three individuals (6%) had TBI values that were not applicable as they had undergone an amputation of the great toe of the affected limb. The chi-square statistic is 4.3046, and the p value is 0.03801, indicating that the results are statistically significant at p < 0.05. The mean TBI was 0.7, with a standard deviation of 0.12, ranging from 0.48 to 0.88.

**Table 6:** Outcome of the ulcer after three months with TBI TBI: toe brachial index

TBI	Improved outcome, n (%)	Unchanged outcome, n (%)	Total, n (%)
≥0.65	21	10	31 (62)
<0.65	16	0	16 (32)
Not applicable	3	0	3 (6)
Total	50	10	100
Mean	0.7		
Standard deviation	0.12		
Maximum	0.88		
Minimum	0.48		

In Table 7, a chi-square statistic of 34.8846 and a very low p value (<0.00001) indicate a highly significant association between TBI and the Doppler findings.

**Table 7:** Association of TBI with Doppler findings TBI: toe brachial index

TBI	Monophasic, n (%)	Biphasic, n (%)	Triphasic, n (%)	Total, n (%)
≥0.65	0 (0)	7 (14)	24 (48)	31 (62)
<0.65	14 (24)	1 (2)	1 (2)	16 (32)
Not applicable	1 (2)	1 (2)	1 (2)	3 (6)
Total	15 (30)	9 (18)	26 (32)	50 (100)

In Table 8, 50 individuals underwent various treatments for their ulcers. Overall, 40 individuals (80%) showed improved outcomes, while 10 (20%) had unchanged outcomes after necessary management.

**Table 8:** Outcome of ulcer after three months based on treatment AKA: above knee amputation; BKA: below knee amputation; STSG: split thickness skin grafting

Treatment, n (%)		Improved outcome, n (%)	Unchanged outcome, n (%)	Total, n (%)
AKA		1 (2)	1 (2)	1 (2)
Angioplasty followed by 11 (22)	BKA	7 (14)	1 (2)	8 (16)
	Debridement	1 (2)	0 (0)	1 (2)
	Lisfranc's amputation	1 (2)	0 (0)	1 (2)
	Transmetatarsal amputation	1 (2)	0 (0)	1 (2)
BKA		2 (4)	0 (0)	2 (4)
Debridement		8 (16)	7 (14)	15 (30)
Debridement and skin grafting		1 (2)	0 (0)	1 (2)
Debridement followed by STSG		12 (24)	0 (0)	12 (24)
Ray's amputation		1 (2)	0 (0)	1 (2)
Regular dressings		4 (8)	3 (6)	7 (14)
Total		40 (80)	10 (20)	50 (100)

## Discussion

The European Study Group on Diabetes and the Lower Extremity identified independent predictors of delayed healing. These included male sex, older age, larger ulcer size, heart failure, inability to stand or walk without assistance, end-stage renal disease, peripheral neuropathy, and PAD [8].

According to our study, the age group between 41 and 50 years had the highest frequency of DFUs, accounting for 48% of the total sample. In a study by Oyibo et al. in 2001, the patient's age did not affect the outcome of the ulcer. Our study also supports this study where the patient's age had no effect on the outcome of the ulcer [9]. A study conducted by Marzoq et al. in 2019 showed statistically significant associations between DFU healing rates and the patients' age; however, this study does not corroborate our study [10].

In our study, we evaluated the outcomes of the ulcer based on gender, with 46% females and 54% males included. In our study, there was no statistical significance between gender and the outcome of the ulcer, which corroborates the study conducted by Oyibo et al., where the patient's gender has no effect on the outcome of the ulcer [9]. However, a study conducted by Khanolkar et al. in 2008 states that the male gender is a risk factor for the development of ulcers, which did not corroborate with our study [11].

Vasculopathy is the main risk factor for major limb amputation, and it should not be overlooked. A noninvasive arterial Doppler scan and a simple clinical assessment of peripheral pulses can help identify this issue early on [12].

Our study analysis indicates a significant association between the history of intermittent claudication and the Doppler findings. A study conducted by Oduola-Owoo et al. in 2022 concluded that patients with symptoms of intermittent claudication have lower extremity PAD, which is common in T2DM and has a higher prevalence in Doppler scans compared with ABPI values [13].

Our study analysis also indicates a significant association between the history of smoking and the Doppler findings. In a 2009 research, Rahman et al. deduced that males in the majority smoke cigarettes, which impede blood flow by increasing vasospasm, blood viscosity, and hypercoagulability, thus predisposing to ischemic foot ulceration. Doppler ultrasound imaging was used to assess the degree of vasculopathy in individuals with diabetic foot disease [14]. Another study conducted by Armstrong et al. states that smoking could commonly contribute to the increased prevalence of peripheral arterial occlusive disease in diabetics [5]. These studies support our study where there is a significant association between the history of smoking and Doppler findings of the affected limb suggestive of vasculopathy.

In the current study, there is a statistically significant correlation between the presence of other comorbidities and the Doppler findings. In our study, 24% of individuals were hypertensive. Armstrong et al. state that a history of hypertension can contribute to the increased prevalence of peripheral arterial occlusive disease in diabetics [5]. Studies conducted by Khanolkar et al. in 2008 demonstrated that the presence of comorbidities could be a risk factor for the development of an ulcer in diabetics [11].

In 2018, Khan et al. concluded that Doppler ultrasonography (dUSG) is a low-cost, easy-to-use, noninvasive technique. The prevalence of diseases and risk factors (such as DM, dyslipidemias, smoking, and a sedentary lifestyle) is rising, which is contributing to the rise in the symptoms of peripheral vascular disease. The study showed an association of significant significance between NIU and smoking, hypertension, and a longer duration of diabetes. The study concluded that prompt management of peripheral vascular disease is cardinal to avert the development of NIU [15].

Identifying the vasculopathy and other macrovascular consequences of DFU is essential by comparing imaging results with clinical symptoms. As a result, we took notice of the cross-sectional study conducted in 2007 by Andersen and Roukis, wherein a clinical diagnosis was established based on the patient's signs and symptoms and corresponded with the diagnosis established using the results of the duplex scan. The study's conclusions show that there was a considerable degree of concordance between duplex and clinical diagnosis. According to this, a duplex scan may be useful for confirming a clinical diagnosis, pointing out other diagnoses, or identifying diseases connected to the clinical diagnosis [16].

A study conducted by Donnelly et al. in 2000 states that ABPI values can be used as an initial diagnosis for PAD [17]. Diabetic patients with vasculopathy can be diagnosed using a variety of techniques, particularly if they are suspected of having lower limb ischemia or pregangrenous alterations. Patients may present asymptomatically in certain cases, but further tests and clinical examination (lack of distal pulses) demonstrate ischemia. A study conducted by Tunçcan et al. in 2012 stated that there was no statistically significant correlation between the abnormal findings in the dUSG and the severity of the wound. However, patients with dUSG pathology were four times more likely to experience a bad outcome, which included amputation. For this reason, all diabetic patients should have a dUSG test to determine whether they are at risk of amputation. This study corroborated our study, where patients with abnormal Doppler findings had poorer outcomes [18].

In 2019, Fareed et al. conducted a study aiming to demonstrate a significant association between the ankle-brachial index and the DFUs in patients who underwent Doppler evaluation [3]. Our study also showed a statistically significant correlation between the ulcer's outcome after three months and Doppler findings regarding ABPI values.

The current study analyzed the impact of TBI on ulcer outcomes, and the results are statistically significant. A highly significant association between TBI and the Doppler findings was also seen. A study by Stoekenbroek in 2015 states that diabetes was not associated with larger differences between ankle and toe pressures among patients with critical limb ischemia. Therefore, the TBI does not allow for earlier detection of ischemia in diabetes [19]. This study does not support our study as there is a significant statistical correlation between TBI and the outcome of the ulcer and Doppler findings in our study.

The decision-making process for treating DFUs, including stent placement, bypass surgery, or amputation, might be aided by the diagnosis of vasculopathy. According to a study done in 2024 by Rümenapf et al., a vascular examination is essential for DFUs and PAD. When it comes to PAD patients, stage-dependent revascularization is crucial, particularly for individuals with critical limb-threatening ischemia [20]. This study supports our study, as revascularization was not done for all patients, and a multidisciplinary approach was used to treat DFUs.

According to research by Frykberg and Banks, treating foot ulcers requires an interdisciplinary team approach to managing comorbidities and early detection [21]. According to a study by Yazdanpanah et al. in 2015, a multidisciplinary team should be used to optimize the therapy of DFUs since wound care calls for a comprehensive approach [22]. Out of 50 cases, 80% showed an improvement in the ulcer, and in 20% of cases, the ulcer characteristics remained unchanged.

Our study highlights the importance of diagnosing vasculopathy and NIUs in DFUs and identifies several risk factors associated with them. Integrated routine assessment of ABPI and TBI should be included in DFU management protocols to guide treatment decisions and monitor response to therapy; however, further research is warranted.

Limitations

The study's limitations were its short duration and sample size. CT Angiography was not done for all patients, and dUSG was done by a single radiologist, which could introduce bias in the study.

## Conclusions

This study underscores that the prevalence of smoking and intermittent claudication and necessitates comprehensive management strategies addressing both the vascular health and lifestyle modifications in a diabetic individual. ABPI and TBI values played a crucial role in predicting and managing ulcer healing outcomes, highlighting the importance of vascular assessment in treatment planning. Continued research and clinical innovation is essential to further refine treatment strategies and mitigate the significant burden of DFUs on both patients and healthcare systems.
